# Association of *Fasciola gigantica* Co-infection With Bovine Tuberculosis Infection and Diagnosis in a Naturally Infected Cattle Population in Africa

**DOI:** 10.3389/fvets.2018.00214

**Published:** 2018-09-06

**Authors:** Robert F. Kelly, Rebecca Callaby, Nkongho F. Egbe, Diana J. L. Williams, Ngu Ngwa Victor, Vincent N. Tanya, Melissa Sander, Lucy Ndip, Richard Ngandolo, Kenton L. Morgan, Ian G. Handel, Stella Mazeri, Adrian Muwonge, Barend M. de C Bronsvoort

**Affiliations:** ^1^The Roslin Institute, Royal (Dick) School of Veterinary Studies, University of Edinburgh, Midlothian, United Kingdom; ^2^Royal (Dick) School of Veterinary Studies, University of Edinburgh, Midlothian, United Kingdom; ^3^Microbiology and Parasitology Unit, Faculty of Allied Medical Science, University of Calabar, Calabar, Nigeria; ^4^Veterinary Parasitology, Institute of Infection and Global Health and School of Veterinary Science, Liverpool, United Kingdom; ^5^School of Veterinary Medicine and Sciences, University of Ngaoundere, Ngaoundere, Cameroon; ^6^Cameroon Academy of Sciences, Yaoundé, Cameroon; ^7^Tuberculosis Reference Laboratory Bamenda, Hospital Roundabout, Bamenda, Cameroon; ^8^Laboratory of Emerging Infectious Diseases, University of Buea, Buea, Cameroon; ^9^Laboratoire de Recherches Vétérinaires et Zootechniques de Farcha, N'Djamena, Chad; ^10^Institute of Ageing and Chronic Disease and School of Veterinary Science, University of Liverpool, Neston, United Kingdom

**Keywords:** bovine tuberculosis, *M. bovis*, co-infection, *F. gigantica*, fasciolosis, Cameroon, diagnostic tests, interferon-γ

## Abstract

Bovine tuberculosis (bTB), caused by *Mycobacterium bovis*, remains a major livestock and public health problem in both high and low-income countries. With the current absence of an effective vaccine, control in cattle populations is reliant on regular testing and removal of positive animals. However, surveillance and control are hampered by imperfect diagnostic tests that have poorly described properties in naturally infected populations. Recent research in cattle co-infected with the temperate liver fluke, *Fasciola hepatica*, has raised concerns about the performance of the intradermal skin test in high fluke incidence areas. Further, recent studies of parasitic co-infections have demonstrated their impact on Th1 and Th2 responses, concurrent disease pathology and susceptibility to mycobacterial infections. Here we report for the first time the association of co-infection with the tropical liver fluke, *Fasciola gigantica*, with the presence of bTB-like lesions and the IFN-γ response in naturally infected African cattle. After adjusting for age and sex we observed a complex interaction between fluke status and breed. Fulani cattle had a higher risk of having bTB-like lesions than the mixed breed group. The risk of bTB-like lesions increased in the mixed breed group if they had concurrent evidence of fluke pathology but was less clear in the coinfected Fulani breed. Further, we observed a slight decline in the IFN-γ levels in fluke infected animals. Finally we explored factors associated with IFN-γ false negative results compared to the presence of bTB-like lesions. Fulani cattle had a higher risk of having a false negative result compared to the mixed breed group. Further, the mixed breed cattle had an increased risk of being false negative if also co-infected with fluke. Interesting, as with the risk of bTB-like lesions, this association was less clear in the Fulani cattle with weak evidence of a slight decrease in risk of having a false negative test result when fluke pathology positive. This interesting interaction where different breeds appear to have different responses to co-infections is intriguing but further work is needed to confirm and understand more clearly the possible confounding effects of different other co-infections not measured here, breed, management or exposure risks.

## Introduction

In natural populations, individuals are usually infected with multiple pathogens, also known as “co-infections,” rather than single infections (1). In the presence of multiple co-infections, the immune response observed to an individual pathogen, across a population, is variable. This has been shown to depend on the combination of infections and their differing interactions with the host immune system and other infections (2). Like many infectious diseases, *Mycobacterium bovis* infection has been studied in isolation until relatively recently. Co-infections with *Fasiola hepatica* have been implicated as a potential reason for poor bTB diagnostic test performance and disagreement between tests (3). More specifically, co-infections with *F. hepatica* have been shown to down-regulate the Th1 responses (with a resultant dampening of the IFN-γ response), with subsequent predominance of Th2 responses, in order for the parasite to survive and reproduce (4–8).

Bovine tuberculosis (bTB), caused by the bacterium *M. bovis*, is both a major veterinary and public health disease of cattle and other livestock. It is an important zoonosis (9, 10) causing pulmonary and extra-pulmonary disease in people and is responsible for an estimated 3% of human tuberculosis globally (11) amounting to an estimated 147,000 zoonotic cases per year, of which 70,000 are in sub-Saharan Africa (www.who.int/tb/areas-of-work/zoonotic-tb/en/). In many high-income countries, such as the United Kingdom and New Zealand, compulsory bTB “test and slaughter” programs coupled with compensation have been successful in reducing transmission of *M. bovis* in livestock populations (12–14). Diagnostic testing involves detection of immune responses in the early stages of infection, such as dominant Th1 responses (15), to remove bTB positive animals as soon possible. Ante-mortem diagnostic tests, such as the single intradermal comparative cervical test (SICCT) or the interferon-γ (IFN-γ) assay, are based on detecting the Th1 immune response to *M. bovis* (16). However, the variable sensitivity of the SICCT (55.1-93.5%) and the IFN-γ assay (73-100%), which rely on detecting this Th1 response, particularly in late stage disease when a Th2 immune response dominates, can lead to false negative cattle persisting within the population (17) resulting in continuing transmission and larger outbreaks.

Although the co-infection relationship has yet to be fully elucidated, various studies have demonstrated that *F. hepatica* co-infection is associated with a reduced Th1 immune response (3) and a reduced mycobacterial burden (18), which potentially leads to the underestimation of bTB prevalence (19). This is particularly important when using the IFN-γ assay to detect bTB positive cattle. IFN-γ is a cytokine which is produced as part of the Th1 immune response to *M. bovis* infection (20). It has been demonstrated that *F. hepatica* infections down-regulate IFN-γ pro-inflammatory cytokine responses in favor of Th2 cytokine induction and an IgG1 response (21). When using the IFN-γ assay to detect bTB infected animals, the presence of *F. hepatica* co-infection can lead to a reduction in IFN-γ response below the diagnostic test cut-off leading to false negative results (22). However, the extent of bTB misdiagnosis using the IFN-γ assay in bTB endemic cattle populations co-infected with other *Fasciola* species, remains unquantified. In addition, although similar immune evasion and modulation strategies to *F. hepatica* have been identified in bovine *F. gigantica* infections, the effect of co-infection with *F. gigantica* on bTB immune responses has been minimally investigated (23).

This paper reports the first study of co-infection with *F. gigantica* (23) in bTB infected cattle under natural conditions in a tropical African population. Bovine tuberculosis (24–26) and *F. gigantica* (27–29) infections are endemic in cattle populations in Cameroon and are currently poorly controlled, providing an opportunity to study their interaction within a natural transmission setting. The data used for this analysis were a subset of data that were generated from a larger study of *M. bovis* epidemiology in Cameroonian cattle (26, 29, 30). We describe the association of *F. gigantica* co-infection with the presence of observable bTB-like lesions and diagnostic test results using the IFN-γ assay.

## Methods

### Abattoir cross-sectional study

**Figure 1:**
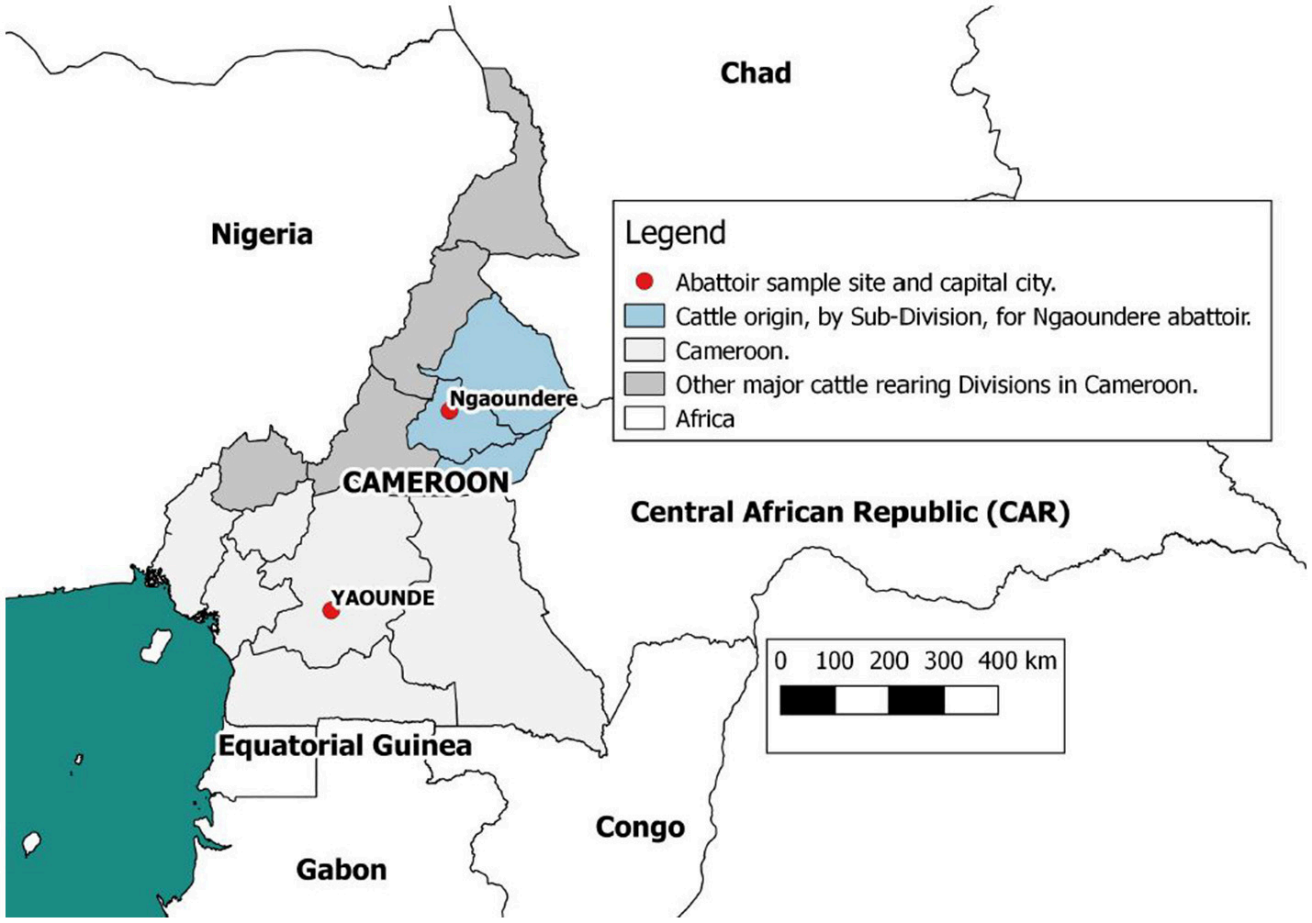
Map of Cameroon showing the location of the Ngaoundere abattoir and the catchment areas of cattle feeding into it. This figure was generated using QGIS 2.2 (www.qgis.org) and shp files obtained from the GADM database of Global Administrative Areas (www.gadm.org).

Data were collected at the Ngaoundere municipal abattoir in the Adamawa Region, a major cattle-producing area of Cameroon (Figure 1). The details of study design and sample collection are reported elsewhere (26) and were based on collection of bTB-like lesion material for culture. In brief, based on previous estimates of bTB-like lesions from the North West Region of Cameroon (31) we assumed a prevalence of lesions of ~5% and calculated a target sample size of ~1000 cattle to ensure recovery of at least 25 isolates assuming a 50% recovery from culture. This would allow the within abattoir prevalence of 5% to be estimated with a precision of ±1.3% at 95% confidence. During sampling, cattle were cast for slaughter by the butchers, after which the research team tagged the animal, collected a heparinized blood sample and recorded animal-level data on owner/butcher, sex, breed as reported by the butcher, dentition score (DS) as an estimation of age (32) and market of origin as reported by the butcher. Post mortem meat inspection was carried out by local Ministry of Livestock, Fisheries and Industrial Agriculture (MINEPIA) inspectors who examined the carcass and offal for presence of granulomatous bTB-like lesions and evidence of liver damage/cirrhosis. Once identified by the veterinary inspectors, the research team collected up to 3 macroscopic bTB-like lesions from different anatomical sites per animal into sterile 25ml universal tubes using forceps and scalpel blades. Lesion grades were also recorded following identification and tissue samples taken (33, 34). Matching numbered tags issued by the research team were used to link animal data, blood samples, meat inspection of the carcass, offal (including the liver) and head. In addition to the tissue samples for culture from animals with lesions, a number of animals classed as non-lesioned by the meat inspectors were randomly sampled (using random number generator www.Random.org) and a single retropharyngeal lymph node per animal collected for culture as controls.

Tissue samples (lesioned lymph nodes) were stored in the vapor phase in liquid nitrogen dry shippers (Taylor-Wharton) and shipped to the Tuberculosis Reference Laboratory (TBRL) Bamenda. Upon arrival at the TBRL the samples were stored at -80°C until processed. Heparinised blood samples were stored in a coolbox at the abattoir (ranging between 10°C to 26°C) and then taken to the lab and kept at room temperature prior to being stimulated in the IFN-γ assay (Bovigam®) described below. Animal data recorded on paper in the abattoir, was transferred to a relational Microsoft Access database where the results could be linked back to individual animals.

### Diagnostic tests

#### *Fasciola* pathology at meat inspection

All carcases were inspected for evidence of *F. gigantica* infection by MINEPIA meat inspectors. The meat inspectors examined the liver systematically to identify gross pathology associated with *Fasciola* infection by slicing down the common bile duct with an additional 1–2 slices through the liver parenchyma. Once an animal was identified to have gross fasciolosis related pathology, the liver was graded by a member of the research team and scored 0–3 (35). A score of 0 = no visible pathology; 1 = low grade pathology with minimal damage to the parenchyma of the liver through migratory fibrotic/ cirrhotic tracts from the parasite, thickening of bile ducts with a few *F. gigantica* parasites noted in bile ducts; 2 = moderate grade pathology with *F. gigantica* species parasites found in the bile ducts and up to approximately half the liver having evidence of fibrosis/ cirrhosis; 3 = severe grade pathology with the majority of the liver is noted to have extensive fibrosis/ cirrhosis without having to cut the surface of the liver. For this analysis the score was converted into a presence (positive) or absence (negative) of *F. gigantica* pathology for subsequent analysis.

#### Mycobacterial culture and typing

The tissue samples were prepared and cultured as previously described (26) following the World Organization of Animal Health (OIE) guidelines with minor modifications. Briefly, samples were processed, inoculated into a Mycobacterial Growth Indicator Tubes (MGIT) and incubated for 8 weeks on the BACTEC MGIT 960 automated culture system (Becton, Dickinson and Company, 1 Becton Drive, Franklin Lakes, NJ, USA) following the manufacturer's instructions. A further 2 cultures were prepared by inoculating 0.1 ml (2 drops) of prepared sample onto each of two Lowenstein Jensen (LJ) slopes (one supplemented with pyruvate and the other with glycerol). These were observed weekly for up to 12 weeks. A smear was made with 3% formal saline from any observed growth on the LJ media and any MGIT indicated positive tube. The smears were heat-fixed, stained by the Ziehl-Neelsen (ZN) method (36) and microscopically observed for the presence of acid fast bacilli (AFB). All acid fast bacilli were typed using the Hain GenoType® MTBC assay and GenoType® Mycobacterium CM/AS kit (Hain Lifescience®,GmbH, Nehren, Germany) (26). Animals were classed as confirmed bTB cases (as opposed to having a bTB-like lesion) if one or more lesions were positive by one or more culture methods confirmed by the Hain Genotype® test.

#### Interferon-gamma assay

The IFN-γ ELISA (Bovigam®) was conducted as per published protocol (37, 38). Briefly within 6–12 h of collection three aliquots of heparinised blood, per animal, were incubated with either avian PPD, bovine PPD (Prionics® Lelystad Tuberculin PPD) or PBS for 24 h at 37°C in a portable polystyrene egg incubator (http://www.theincubatorshop.co.uk) run in the field. Following incubation samples were centrifuged at 300g for 10 minutes, the plasma was aliquotted and stored at −20°C in a portable travel freezer (Waeco CF50 12V/240 fridge freezer)). Plasma samples were transported at −20°C to the LEID and the IFN-γ ELISA was conducted as per the published protocol. The acceptable averaged negative bovine OD value was < 0.130 and positive bovine control was >0.700. Animals with a bovine stimulated sample optical density of ≥0.1 above that of the avian PPD sample were classified as test positive and interpreted as the animal being infected with *M. bovis*.

### Statistical analysis

The proportions of cattle with bTB-like lesions, a positive IFN-γ result and liver fluke pathology were calculated and various co-infection definitions were explored using *Fasciola* pathology and one of the bTB outcomes (positive IFN-γ, bTB-like lesion or *M. bovis* culture positive).

Multivariable logistic regression (MLR) models were developed to explore the association between fluke infection and bTB status using a number of different definitions including IFN-γ, bTB-like lesion and *M. bovis* culture results. Animal-level explanatory variables (breed, dentition score and sex) were always included in the models as fixed effects to control for confounding. Model selection was based on the AIC and the best model was selected using the lowest AIC and ΔAIC (39). MLR models were constructed using the *brglm* function in the *brglm* package (40) with AIC and ΔAIC calculated using the *modavg* function from the *AICcmodavg* package (41). Predicted probabilities and their standard errors were calculated from each model for specific covariate patterns using the *predict* function and used to produce 95% confidence intervals for plotting.

Some variables were simplified due to small numbers of observations in some categories. The dentition score (DS) was simplified from number of permanent teeth to a binary age category based on the approximate relationship between age and eruption of permanent incisors in cattle. DS <2 was categorized as “ <3 years old” and DS of ≥2 was categorized as “≥3 years old.” The breed variable was simplified to “mixed” (collapsing mixed breed, where the butchers were unsure of the breed cross and Gudali to a single category) or “Fulani” (collapsing Red and White Fulani to a single category).

## Results

### Summary statistics

During a 4 week period in August 2013, 935 cattle were examined at the Ngaoundere abattoir. The details of bacterial culture results have been presented elsewhere (26). A total of 173 records were dropped due to missing data (at random) due to the hectic nature of the sampling in the abattoir environment which resulted in occasional failure to collect a blood sample or link samples to a carcass. A further 30 animals from one day were dropped from this analysis due to missing IFN-γ results giving a final dataset of 732 animals.

**Figure 2:**
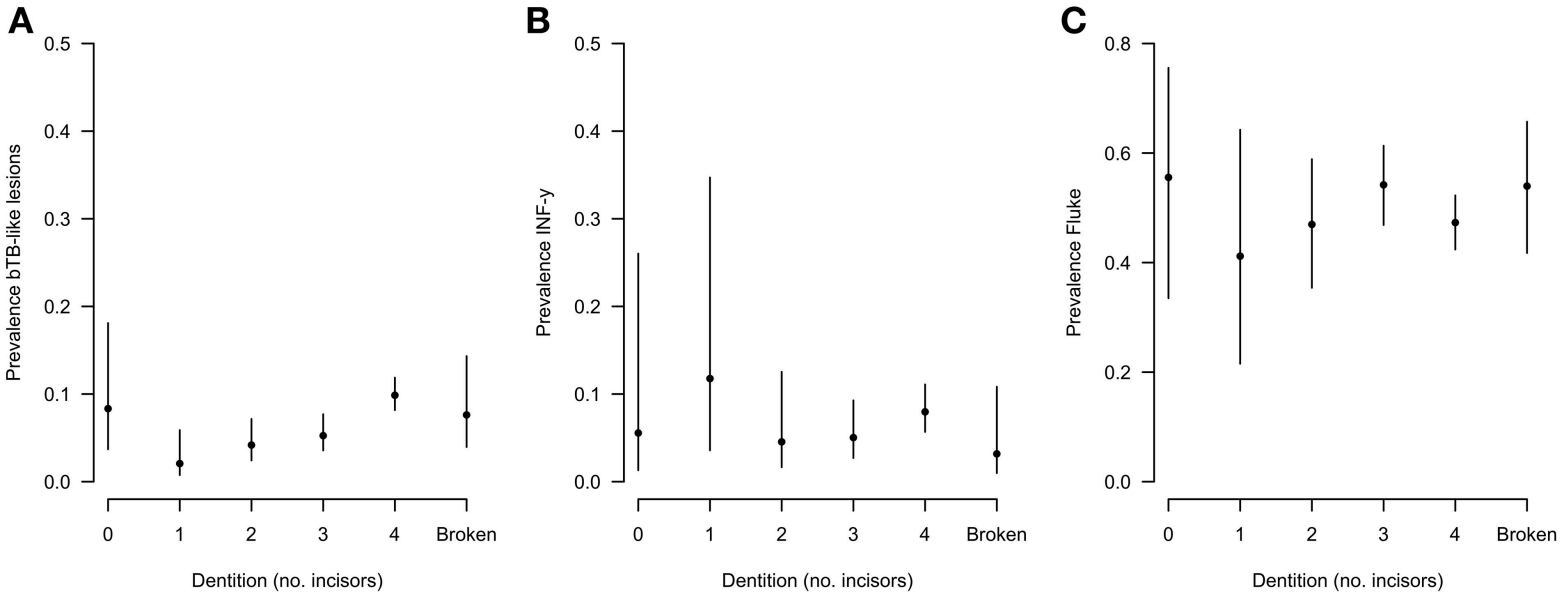
Distribution of **(A)** lesion, **(B)** fluke, and **(C)** IFN-γ prevalences based on animal dentition for Ngaoundere abattoir (*n* = 732).

**Figure 3:**
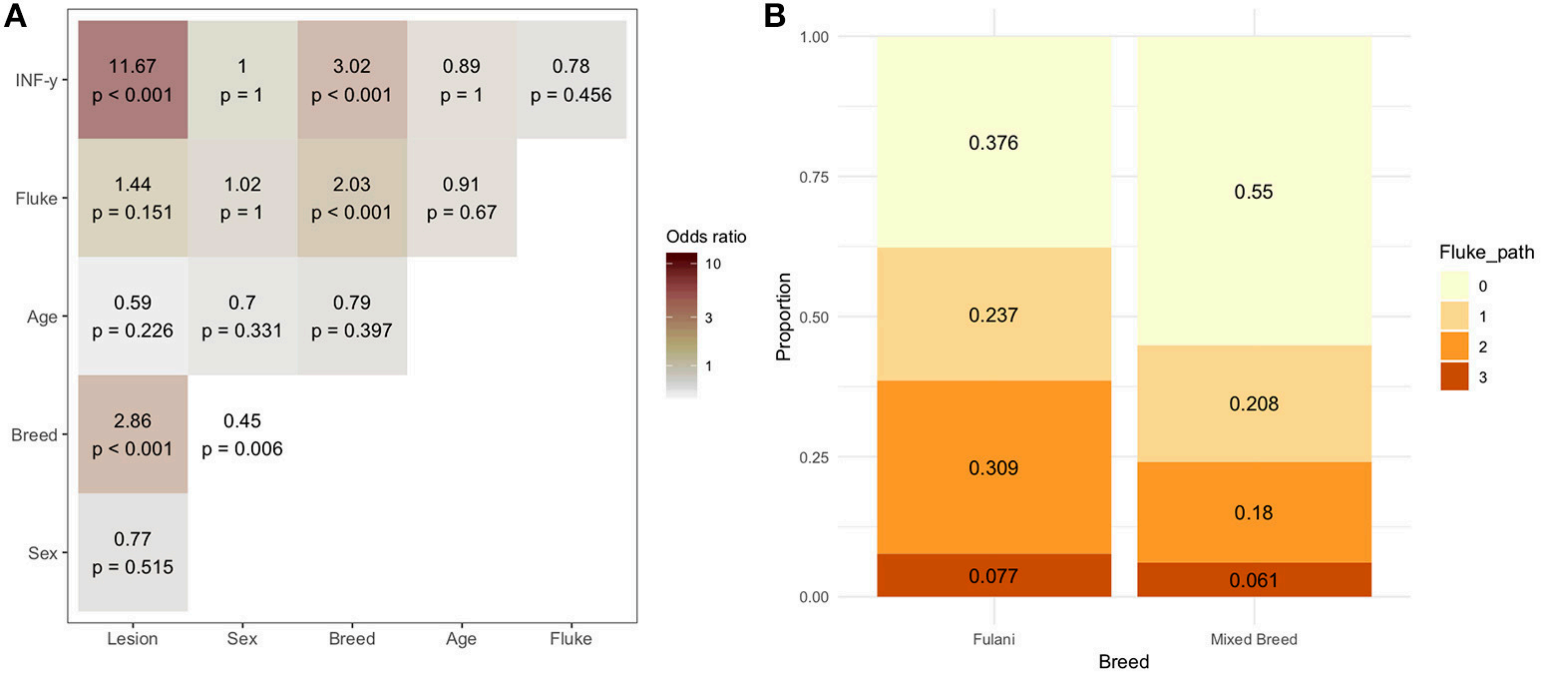
**(A)** Bivariable odds ratios and *p*-values for pairwise associations of variables of interest for inclusion in multivariable models. **(B)** Proportion of Fulani (*n* = 194) and mixed breed (*n* = 538) classified cattle sampled in Ngaoundere abattoir by *F. gigantica* pathology score. Legend: Lesion, lesion status (positive/negative); sex, male/female; Breed, Fulani/mixed; Age, < 3 years/≥3 years; Fluke, fluke pathology status (positive/negative); IFN-γ, IFN-γ test positive/negative.

During the sampling period 10.7% (78/732) of animals had visible bTB-like lesions observed. The proportion of animals with evidence of liver fluke infection was 49.6% (363/732) and the proportion of animals positive by the IFN-γ assay was 6.6% (48/732). The distribution of the prevalances of bTB-like lesion, liver fluke pathology and IFN-γ based on animal dentition (as a measurable proxy for age) are given in Figure 2. Exploratory bivariate relationships between the variables of interest (age, breed, sex, lesion status, fluke status and IFN-γ status) were checked prior to inclusion in the multivariable models (Figure 3A). Breed and fluke status were strongly associated with presence of visible bTB-like lesions and breed was strongly associated with IFN-γ status. There was an association between breed and the ordinal distribution of fluke pathology scores (χ^2^ test p-value <0.001) but the odds ratios did not change across pathology scores above zero so the effect is captured by collapsing the fluke score into a binary variable (Figure 3B).

### Association of *F. gigantica* co-infection with bovine tuberculosis-like lesions

A multivariable logistic regression (MLR) model of the association between visible lesion and fluke status was developed (Table 1). This suggests a complex interaction between breed and *F. gigantica* pathology status with the probability of observing visible TB-like lesions in cattle in this setting. Fulani cattle had a higher risk of having observable bTB-like lesions than the mixed breed group. However, the risk in the mixed breed group increased if they also had fluke pathology. This association ith fluke pathology was less clear in Fulani cattle. For example, an adult, female, mixed breed animal, that had no fluke pathology had a ~5.2% (95% CI: 2.5–7.8) probability of having a TB-like lesion compared to ~27.7% (95% CI: 16.8–38.4) for a Fulani animal (Figure 5A). The presence of fluke increased this risk in mixed breed animals to ~12.0% (95% CI: 7.7–16.3) while the risk declined (although the evidence is weaker) in Fulani cattle to ~15.6% (95% CI: 8.8–22.2). The age and sex terms were included to control for confounding but do not appear to be important for this model.

**Table 1:** Multivariable logistic regression model for the presence of TB lesions at slaughter (*n* = 732).

**Variable**	**Levels**	**Odds ratio**	**95% CI**
Sex	Female	1	
	Male	1.17	0.50–2.47
Age	≥3 years	1.00	
	<3 years	0.60	0.25–1.26
Breed	Mixed	1	
	Fulani	7.00	3.36–14.95
Fluke	Negative	1	
	Positive	2.51	1.32–4.98
Breed*Fluke		0.19	0.07–0.51

Key: Lesion, TB lesion result (Positive or negative); Sex, Sex of cattle (Male or Female); Age, Age of cattle by dentition score (< 3 years or ≥3 years); Fluke, F. gigantica pathology score; Breed, Breed of cattle (Mixed breed or Fulani breed);

* *Interaction between variables*.

### Impact of *F. gigantica* co-infection on IFN-gamma responses

**Figure 4:**
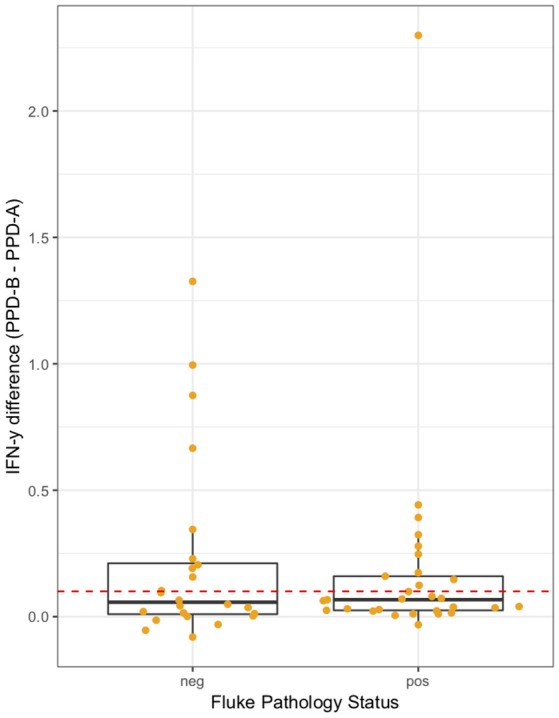
IFN-γ response (PPD-B minus PPD-A) stratified by *F. gigantica* pathology status (neg, no pathology; pos, evidence of fluke pathology) in the subset of *M. bovis* culture positive cattle (*n* = 53). Individual cattle; Orange circles. Dashed red line IFN-γ recommended test cut-off of 0.1.

The association between IFN-γ result and *F. gigantica* pathology status was investigated. The raw PPD-B minus PPD-A difference in ELISA OD readings were explored in the subset of animals which were confirmed *M. bovis* culture positive (n = 53). The raw difference is plotted, stratified by *F. gigantica* pathology status in Figure 4 where there is some evidence of a dampening of the IFN-γ response with a smaller variance in the fluke pathology positive group. When the outlying high value for the fluke pathology positive group is removed the variances are statistically significantly different (Mann-Whitney test *p* < 0.001, *n* = 52).

**Figure 5:**
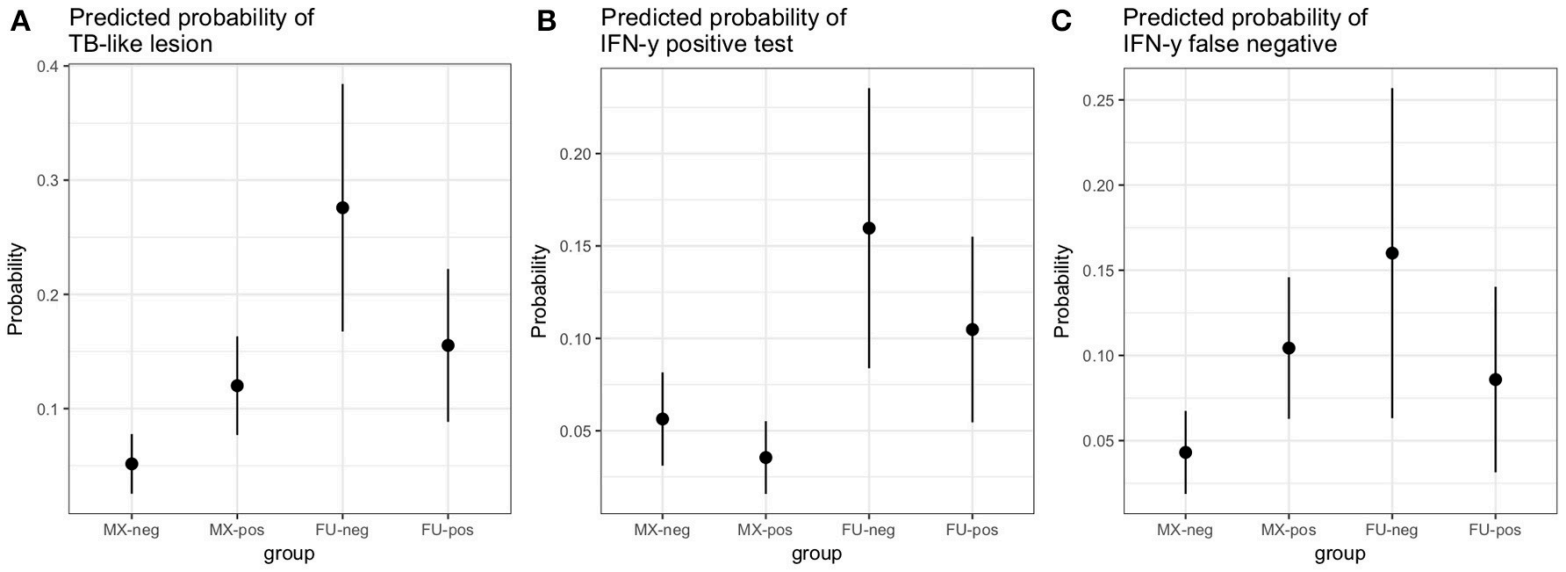
Predicted probabilities for an adult female animal by breed (MX, mixed breed; FU, Fulani) and fluke status (neg, no fluke pathology; pos, evidence of fluke pathology) based on the logistic regression models described in the results section.

A multivariable linear regression model of the raw PPD-B minus PPD-A difference was developed (with the outlying value dropped) and after accounting for age, sex and breed there was a small mean decrease in the difference of −0.02 (−0.04 to 0.00) in OD value in the fluke pathology positive animals (Table 2). A multivariable logistic regression model for IFN-γ binary test status and fluke pathology status was also developed including age, breed and sex as potential confounders (Table 3). Both these regression models give some weak support for an association between the IFN-γ result and *F. gigantica* pathology status, with fluke pathology positive cattle more likely to have a lower OD value difference and to be IFN-γ negative. The association using the binary test results suggest an adult, female, mixed breed cow had a ~5.6% (95%: 3.1–8.1) probability of testing positive which dropped to ~3.5% (95%: 1.7-5.6) if infected with liver fluke compared to a Fulani, adult, female animal which had a ~16.3% (95%: 8.7–24.0) of testing positive which dropped to ~11.1% (95%: 5.9–16.2) if fluke pathology positive (Figure 5B).

**Table 2:** Multivariable regression model for the raw IFN-γ PPD-B minus PPD-A difference (*n* = 731).

**Variable**	**Levels**	**Coef**	**95% CI**
Sex	Female	1	
	Male	–0.01	–0.46 to 0.02
Age	≥3 years	1	
	<3 years	–0.01	–0.31 to 0.02
Breed	Mixed	1.00	
	Fulani	0.02	0.13 to 1.46
Fluke	Negative	1	
	Positive	–0.02	–1.92 to 0.01

*Key: IFN-γ, IFN-γ assay result (PPD-B - PPD-A); Sex, Sex of cattle (Male or Female); Age, Age of cattle by dentition score (< 3 years or ≥3 years); Fluke, F. gigantica pathology score; Breed, Breed of cattle (Mixed breed or Fulani breed)*.

**Table 3:** Multivariable logistic regression model for the raw IFN-γ PPD-B minus PPD-A difference (n = 731).

**Variable**	**Levels**	**Odds ratio**	**95% CI**
Sex	Female	1	
	Male	0.91	0.28–2.29
Age	≥3 years	1	
	<3 years	0.99	0.38–2.22
Breed	Mixed	1.00	
	Fulani	3.18	1.73–5.85
Fluke	Negative	1	
	Positive	0.62	0.33–1.13

*Key: IFN-γ, IFN-γ assay result (Positive or negative); Sex, Sex of cattle (Male or Female); Age, Age of cattle by dentition score (< 3 years or ≥3 years); Fluke, F. gigantica pathology score; Breed, Breed of cattle (Mixed breed or Fulani breed)*.

### Factors associated with a false negative IFN-γ test result when compared to TB-like lesion

Using the subset of results where the IFN-γ test result was negative (*n* = 684) a new variable was generated where a IFN-γ test result was classified as a false negative if there was an observed bTB-like lesion (*n* = 54) and true negative if there was no lesion observed (*n* = 630). A multivariable logistic regression analysis to explore the association with fluke pathology was conducted including sex, age and breed as confounders. In addition, for this model the presence or absence of non-tubercular mycobacteria (NTM), based on the Haines typing from cultured samples from lesions, was also included as a known potential confounder for being lesion positive but test negative. The final model is given in Table 4. Again the best fitting model includes an interaction between fluke pathology status and breed. The baseline probability of being a false negative IFN-γ test result in adult, female, non-tubercular mycobacterium negative (NTM), fluke pathology negative mixed breed cows was ~4.3% (95%: 1.9–6.7) which increased to ~10.4% (95%: 6.3–14.5) if they had fluke pathology. In comparison IFN-γ test negative Fulani cattle had a ~16.0% (95%: 6.3–25.6) probability of being a false negative which declined to ~8.5% (95%: 3.1–14.0) if fluke pathology positive (Figure 5C). An NTM infection also was also associated with a large increased risk of giving a false negative IFN-γ result.

**Table 4:** Multivariable logistic regression model (*n* = 684) for being IFN-γ false negative result conditioned on having a negative IFN-γ test result and using observable TB-like lesions as the true state (gold standard).

**Variable**	**Level**	**Odds ratio**	**95% CI**
Sex	Female	1	
	Male	0.97	0.31–2.46
Age	≥3 years	1	
	<3 years	0.68	0.24–1.57
NTM	Negative	1	
	Positive	15.29	3.32–75.7
Breed	Mixed	1	
	Fulani	4.23	1.68–10.44
Fluke	Negative	1	
	Positive	2.59	1.28–5.49
Breed*Fluke		0.19	0.06–0.64

Key: Lesion, TB lesion result (Positive or negative); Sex, Sex of cattle (Male or Female); Age, Age of cattle by dentition score (< 3 years or ≥3 years); Fluke, F. gigantica pathology score; Breed, Breed of cattle (Mixed breed or Fulani breed); NTM, non-tubercular mycobacterium;

* *Interaction between variables*.

## Discussion

It is well recognized, though still poorly studied, that co-infecting pathogens can have a range of synergistic or antagonist effects. For example, (42) showed African buffalo co-infected with strongyle nematodes had a dampened Th1 response which facilitated bTB invasion and establishment of infection. Interactions between bovine tuberculosis and the temperate liver fluke *F. hepatica* have been shown in the United Kingdom (19, 3) but less is known about the interactions of bovine tuberculosis and the tropical fluke *F. gigantica*. Despite the ecological, genetic and antigen differences between these two species, both species appear to evade and modulate the host immune response to infection (43). Here we have examined the association between both bTB-like lesion occurrence and the Bovigam (*M. bovis specific*) IFN-γ response in a naturally *F. gigantica* infected cattle population in Africa.

The levels of fluke infection were very high in this abattoir population with nearly 50% of animals showing liver pathology consistent with fluke infections and/or adult fluke identified in the bile ducts. Previous studies have identified an association with visible bTB-like lesions and being fluke positive in slaughter cattle populations such as in Zambia (44). Further, in experimental infections in mice it has been shown that mice infected with *M. tuberculosis* have higher bacterial loads and TB lesion pathology when co-infected with the trematode *S. mansoni* (45). It is proposed that the fluke infection suppresses the Th1 response resulting in a reduced level of IFN-γ. In cattle, previous studies have reported that co-infection results in a lower bacterial load but no qualitative or quantitative differences in tuberculous lesions of *M. bovis* (18).

In the present study, we observed a complex interaction between cattle breed and liver fluke pathology status and the presence of visible bTB-like lesions. There is strong support for an increased risk of having bTB-like lesions in Fulani cattle compared to the mixed breed group. In Cameroon, Fulani cattle have been reported to have a higher prevalence of bTB than other breeds (31) but this study was from the Northwest Region. One explanation may be to do with differing responses in different cattle/host genotypes. It has been reported in the UK that Holstein cattle with the INRA111 genotype appeared to be less likely to develop bTB (46). Similarly, in Ethiopia comparing Holstein cattle and indigenous zebu *B. indicus* cattle, researchers found that Holstein cattle were more susceptible (33).

The presence of fluke pathology in the mixed breed group was associated with an increased risk of visible lesions. In Fulani cattle, which are more likely to have bTB-like lesions, co-infection with fluke was not associated with an increased risk but potentially a reduced probability of having visible bTB-like lesions, although the evidence was weak for this association in Fulani cattle. Our study relied on the butchers classification of breed recorded in the hectic environment of the slaughterhouse. There is the possibility that their classification was incorrect in some cases, however, this is more likely to reduce the chances of observing an association. To improve on this we are currently genotyping the subset of cattle from which we collected lymph nodes for culture (both lesioned and the random sample of non-lesioned lymph nodes). Alternatively, the breed association may be due to confounding by other unobserved variables such as differing management between breeds or differences in exposures interacting with different genotypes of *M. bovis*. However, these are more difficult to untangle.

The IFN-γ assay is particularly useful to detect early (1-4 weeks post infection) *M. bovis* infections as part of control programs often in combination with the SICCT (20, 47). Previous studies have demonstrated that *F. hepatica* co-infection can down-regulate IFN-γ responses to *M. bovis* infection (21, 22). In this African cattle population there was weak evidence of a reduced IFN-γ response (reduced variance in the raw PPD-B - PPD-A value) in fluke infected animals conditional on having been bTB culture positive. This reduction in IFN-γ was also weakly observed in the linear regression analysis (on the continuous test result) and in the logistic regression analysis (on the binary result). However, we did observed a moderate association between increased IFN-γ levels and breed, with Fulani cattle having a higher probability of testing positive. Given the higher rates of infection observed in the lesion data, this is not surprising. This represents relatively weak evidence for a decline in test sensitivity in fluke infected animals and increased risk of leaving potential bTB positive animals in a herd. However, the prevalence of bTB was relatively low in this relatively small study, meaning the power to detect these effects is less than ideal and further larger studies are needed to confirm these findings.

In order to further explore this potential decline in test sensitivity, we looked at the subset of IFN-γ test negative animals (based on the binary cut-off of 0.1 as recommended by the manufacturers). Using visible bTB-like lesions as the comparison test, which we know from culture results from these cattle is reasonably specific (sp = 69.7%) and sensitive (95.8%), (with a positive predictive value of 65.1% and negative predictive value of 96.6% calculated from (30) for this sample), we looked at factors associated with false negative results in the IFN-γ negative subset of animals. The risk of false negative results was strongly associated with NTM infections. This is to be expected as we know that lesions are an imperfect predictor of *M. bovis* and that a number of these animals with lesions had NTMs based on culture results (30). Interestingly, having accounted for a major source of the disagreement, there again remained a complex interaction between breed and fluke status. In the mixed breed animals the risk of being a false negative increased from ~4% to ~10% consistent with suppression of the Th1 response by fluke infections. Also Fulani cattle had higher rates of false positives compared to the mixed breed group but Fulani cattle with fluke pathology had a drop in risk from ~16% to ~8.5%, although the statistical support for this decline is weak. Again, this may be due to different host genetics, management or exposures and needs further investigation.

More work is needed to understand these interactions between co-infecting pathogens. Variation in immune interaction of the host, with *M. bovis* and *Fasciola gigantica*, at different stages in the pathogenesis of one pathogen may affect the pathogenesis of the other (48, 49). One possible explanation for this complexity may be that we have not accounted for other confounding co-infections. Conducting studies to look at all possible co-infections can become extremely complex, expensive and logistically challenging (50). There have been a number of co-infection studies including nematode infections in buffalo (42) and fluke in cattle (19) with evidence of associations with bTB-like lesions or interference with IFN-γ test results, however, a recent paper failed to find a statistical association with fluke or bovine viral infections (51) in European cattle, although they did find an association with paratuberculosis co-infections, which were associated with an increased probability of observing visible bTB-like lesions.

It is clear that co-infections can have complex impacts on test diagnostics and pathogen invasion and this may have important implications for control programmes. As one bacillus is sufficient to establish *M. bovis* infection within a host (52), leaving any infected animals behind in a control programme has the potential to maintain transmission and there was evidence here that in the mixed breed group at least, fluke infections were associated with an increased risk of a false negative IFN-γ result. Certainly, test and slaughter programs are likely to continue to play their part in bTB control in high income settings and certain wildlife control settings. Mitigating against *Fasciola spp*. co-infections (or other co-infections such as paratuberculosis) or being able to incorporate the likely impact on test performance may improve ante-mortem diagnostic test sensitivity within cattle or wildlife populations.

In conclusion, this study explored the association between co-infection with the tropical liver fluke *F. gigantica* on the pathology and detection of *M. bovis* infections in a natural ecological setting in African cattle. We have shown a complex association between the presence of visible bTB-like lesions in carcasses and the presence of concurrent *Fasciola* infections which appears to be also affected by breed. Furthermore, we have shown that the IFN-γ response may be slightly dampened down in *F. gigantica* infected cattle although further data are needed to confirm this. However, it does appear to be sufficient to increase the false negative risk in the mixed breed group at lest in this population. The reduction in sensitivity of the IFN-γ assay by *Fasciola spp*. co-infection could have profound effects on bTB control and eradication programs as *Fasciola spp*. are present worldwide (53). Given the complexity of determining whether animals are truly *M. bovis* infected, there is a need to develop more subtle and sophisticated algorithms for interpretation of individual animal bTB diagnostic test results that use the raw test readings as well as other animal and related herd variables.

## Ethics statement

This study did not involve the experimental use of any live vertebrate but reports the results of a pre-slaughter blood sampling and post mortem examinations of cattle for bovine tuberculosis carried out by the local veterinary inspectors in a commercial abattoir in Cameroon. Samples for culture were collected from carcases in accordance with best practice guidelines to minimize contamination. Local approval was given by the Head of Epidemiology at the Ministry of Livestock, Fisheries and Animal Industries responsible for supervision of activities at commercial slaughterhouses in each administrative Division. This project and the protocols had ethical approval from the University of Edinburgh Ethical Review Committee (Animal (Scientific Procedures) Act, 1986) (ERC No: OS02-13).

## Author contributions

BB, RK, LN, VT, KM, RN, MS, and NE: conceived and designed the study; RK, SM, NV, NE, and AM: performed the field work; RK, BB, IH, SM, and RC: analyzed the data; RK, BB, IH, SM, MS, and DW: contributed reagents, expertise, materials, analysis tools; RK and BB: wrote the first draft paper; All authors read and contributed to the final draft of the paper.

### Conflict of interest statement

The authors declare that the research was conducted in the absence of any commercial or financial relationships that could be construed as a potential conflict of interest.
